# Infections Following Gender-Affirming Vaginoplasty: A Single-Center Experience

**DOI:** 10.1093/ofid/ofae526

**Published:** 2024-09-11

**Authors:** Radhika Sheth, Apoorva Bhaskara, Haley Brown, Cara D Varley, Amber Streifel, Marissa Maier, Monica K Sikka, Christopher Evans

**Affiliations:** Division of Infectious Disease, Oregon Health & Science University, Portland, Oregon, USA; Division of Internal Medicine, Oregon Health & Science University, Portland, Oregon, USA; Oregon Health & Science University Portland State University School of Public Health, Portland, Oregon, USA; Division of Infectious Disease, Oregon Health & Science University, Portland, Oregon, USA; Oregon Health & Science University Portland State University School of Public Health, Portland, Oregon, USA; Division of Infectious Disease, Oregon Health & Science University, Portland, Oregon, USA; Department of Pharmacy, Oregon Health & Science University, Portland, Oregon, USA; Division of Infectious Disease, Oregon Health & Science University, Portland, Oregon, USA; Department of Infectious Disease, Veterans Affairs Portland Health Care System, Portland, Oregon, USA; Division of Infectious Disease, Oregon Health & Science University, Portland, Oregon, USA; Department of Infectious Disease, Veterans Affairs Portland Health Care System, Portland, Oregon, USA; Division of Infectious Disease, Oregon Health & Science University, Portland, Oregon, USA; Division of Internal Medicine, Oregon Health & Science University, Portland, Oregon, USA

**Keywords:** gender-affirming care, HIV, surgical infections, transgender, UTI

## Abstract

We describe the epidemiology and incidence of infections following gender-affirming vaginoplasty. Urinary tract and surgical site infections were the most common infections with incidences of 17.5% and 5.5%, respectively. We also identified a significant gap in human immunodeficiency virus screening and prescription of preexposure prophylaxis.

## Graphical Abstract

**Graphical Abstract:**
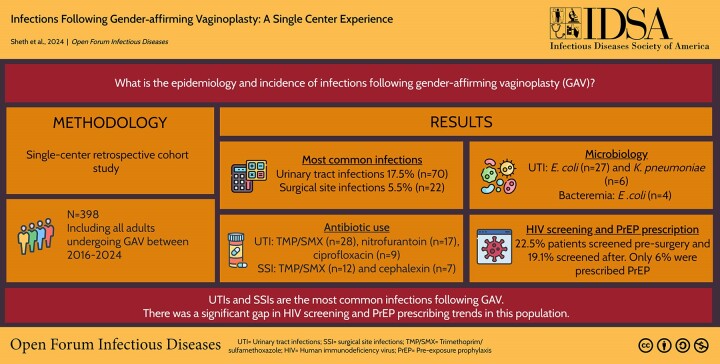
https://tidbitapp.io/tidbits/infections-following-gender-affirming-vaginoplasty-a-single-center-experience-383f8b1f-beaa-4489-93b1-b4dc342c0ae4?utm_campaign=tidbitlinkshare&utm_source=IO

An estimated 1.3 million adults in the United States are transgender [1], and approximately 25% choose to undergo gender-affirming surgery (GAS) [2]. Since the expansion of insurance coverage for GAS, there has been a rise in the number of people seeking GAS procedures [3, 4].

Vaginoplasty involves creation of the vagina and external female genitalia. A penile inversion technique is most commonly used, whereby a neovagina, clitoris, and labia are constructed using primarily penile skin, glans, and scrotal skin, respectively [5, 6]. Alternatively, robot-assisted vaginoplasty uses peritoneum to create the neovaginal apex and augment the depth of the neovagina created with skin [7]. Less frequently, intestinal vaginoplasty may be performed using a segment of the colon to create the neovagina.

Data regarding post-GAS infectious complications remain limited. These knowledge gaps can contribute to less informed stewardship interventions, presurgical counseling, and delays in infection recognition in transgender and gender-diverse populations. We describe the epidemiology and incidence of infections following gender-affirming vaginoplasty (GAV).

## METHODS

### Study Design

This is a retrospective cohort study of adult patients (≥18 years) undergoing GAV at a single tertiary care center in Portland, Oregon, between 2016 and 2023. The patients were identified from a prospective list of GAV maintained by the surgeons at our institution. We describe the incidence, microbiologic features, and treatment of infections following GAV. Patient consent was not applicable to this study, and institutional review board (IRB) approval was obtained (IRB no. STUDY00025773).

### Variables

We reviewed electronic medical records of eligible patients for 6 months following the date of index GAV. We collected human immunodeficiency virus (HIV) screening performed at any site in our health system within a year before and after surgery. We collected patient demographics (age, race, and ethnicity), body mass index, medications (steroids, antibiotics, antiretroviral therapy [ART], and HIV preexposure prophylaxis [PrEP]), surgery and admission dates, and microbiologic data using SAP BusinessObjects Enterprise Business Intelligence Platform 4.2 (SAP America). We collected medical, social, and surgical history via record review.

### Definitions

We used the Centers for Disease Control and Prevention's National Healthcare Safety Network (symptom criteria to define surgical site infections (SSIs) [8]. Two physicians (R. S. and A. B.) reviewed records for documentation of SSI signs (fever, purulent drainage, localized pain, tenderness, wound dehiscence, necrosis, or increasing edema or erythema) occurring within 6 months after GAV. We defined SSI as having ≥2 signs. We recorded urinary tract infections (UTIs) and sexually transmitted infections (STIs), including syphilis and vaginal, urethral, rectal, and pharyngeal chlamydia and gonorrhea. We defined STIs as a positive nucleic acid amplification test result or a reactive rapid plasma regain test with titers consistent with new syphilis diagnosis. We defined UTIs as cystitis or pyelonephritis symptoms (≥1 of the following: dysuria, frequency, suprapubic pain, flank pain, or fever) prompting antibiotic prescription. An independent infectious diseases (ID) physician reviewed a random subset (13%) of patients to confirm the diagnosis. In case of a disagreement, a second ID physician's assessment was obtained.

### Statistical Analysis

We presented categorical variables as frequencies and percentages, and continuous variables as median and interquartile range (IQR). We calculated infection incidence with each patient contributing person-time from GAV until the censor date. Patients were censored at first infection, 6 months, or date of last encounter if lost to follow-up. We also performed univariable logistic regression to identify variables associated with infection. Statistical analyses were performed using RStudio software, version 4.3.2 (RStudio PBC).

## RESULTS

Between 2016 and 2023, a total of 398 patients underwent GAV. Of those, 80% were white and 86.5% non-Hispanic, with a median age (IQR) of 39 (18–79) years (Table 1).

**Table 1.** Patient Characteristics at Baseline

Characteristic	Patients, No. (%)^a^	
	Without Infection (n = 322)	With Infection (n = 76)^b^
Age at time of surgery, median (IQR), y	39 (18–79)	37 (18–71)
Race		
White	259 (80)	59 (78)
Black or African American	7 (2.2)	5 (6.6)
American Indian or Alaska Native	12 (3.7)	…
* Asian*	9 (2.8)	…
Native Hawaiian or Pacific Islander	12 (3.7)	0 (0.0)
* Unknown*	23 (7.1)	5 (6.6)
Combined^c^	…	7 (9.2)
Ethnicity		
Non-Hispanic	283 (88)	58 (76)
Hispanic or Latino	19 (5.9)	6 (7.9)
Unknown	20 (6.2)	12 (16)
BMI, median (IQR)^d^	26.8 (16.1–50.5)	27.7 (16.0–44.6)
Comorbid conditions		
History of diabetes	19 (5.9)	6 (7.9)
History of cardiac disease	12 (3.7)	2 (2.6)
HIV infection	15 (4.7)	1 (1.3)
ART prescribed at time of surgery		
Yes	15 (93.8)	1 (6.3)
No	0 (0.0)	0 (0.0)
Viral load at time of surgery		
* *Detectable	3 (18.8)	0 (0.0)
Undetectable or LLQ	10 (62.5)	1 (6.3)
Unknown	2 (12.5)	0 (0.0)
CD4 cell count at time of surgery		
<200/µL	0 (0.0)	0 (0.0)
201–499/µL	1 (6.3)	0 (0.0)
500–999/µL	6 (37.5)	1 (6.3)
*>1000*	4 (25.0)	0 (0.0)
Unknown	4 (25.0)	0 (0.0)
PrEP use	20 (6.2)	3 (3.9)
History of viral hepatitis		
Hepatitis B^e^	5 (1.6)	2 (2.6)
Hepatitis C^f^	7 (2.2)	3 (3.9)
Immunosuppressant use^g^	6 (1.9)	1 (1.3)
Steroid use 30 d before surgery^h^	3 (0.9)	0 (0)
Prior pelvic surgery	49 (15)	17 (22)
Use of gender-affirming hormone therapy	322 (100)	75 (98.6)
Radiation to pelvis	1 (0.3)	0 (0)
Social history		
Tobacco use		
No history	161 (50)	38 (50)
Current	14 (4.3)	8 (11)
Former	147 (46)	30 (39)
Drug use^i^		
Current	11 (3.4)	2 (2.7)
Former	15 (4.7)	7 (9.3)
Surgical technique		
Penile inversion	282 (88)	50 (60)
Robotic	40 (16)	24 (32)
*Intestinal*	0 (0)	2 (2.6)
Use of extragenital skin graft	49 (15)	8 (11)
Duration of postsurgical urinary catheterization, median (IQR), d	5 (5­5)	5 (5­5)

Abbreviations: ART, antiretroviral therapy; BMI, body mass index; HIV, human immunodeficiency virus; IQR, interquartile range; LLOQ, lower limit of quantification; PrEP, preexposure prophylaxis.

^a^Data represent no. (%) of patients unless otherwise indicated.

^b^Some individuals had multiple episodes and types of infection.

^c^Combined race, for patients with infection, includes Asian, Native Hawaiian, or Pacific Islander; the data for individual categories were suppressed for confidentiality due to small numbers.

^d^BMI calculated as weight in kilograms divided by height in meters squared.

^e^Defined as documented past or current infection with hepatitis B in the medical record, based on hepatitis B serologic findings consistent with present or past infection or current treatment for hepatitis B.

^f^Defined as documented past or current infection with hepatitis C in the medical record, based on hepatitis C serologic findings consistent with present or past infection or current treatment for hepatitis C.

^g^Defined as any immune modulators, chemotherapy, or monoclonal antibody infusions expected to increase the patient's risk of infection—not including steroids.

^h^Including any systemic steroid use in the 30 days before vaginoplasty. This excluded any steroid use during the surgery itself.

^i^Defined as active use or a history of using ≥1 nonmarijuana drug, including both injection and noninjection drug use.

### Surgical Techniques and Prophylaxis

Among the cohort, 381 patients underwent primary vaginoplasty, and 17 underwent revision of the primary surgery. Surgical techniques included standard penile inversion in 332, robotic vaginoplasty in 64, and sigmoid vaginoplasty in 2. Extragenital skin grafts were required in 57 patients. The median postsurgical duration (IQR) for an indwelling urinary catheter was 5 (5–5) days for those with or without infection. Cefazolin was the most common antibiotic for preoperative prophylaxis (96.5%), and cephalexin plus metronidazole was the most common postsurgical antibiotic prophylaxis (92%), typically given for 5 days. In univariable logistic regression, none of the variables reached statistical significance (*P* < .05), including prior pelvic surgery, surgical technique, duration or graft tissue, tobacco use, BMI, post-GAV catheter duration, HIV infection, steroid or other immunosuppressant use.

### Incidence of Post-GAV Infections

We identified an overall infection incidence of 1.25/1000 person-years, with median time to infection (IQR) of 53 (2–162) days after GAV. UTIs were most common (17.5% [n = 70]), followed by SSIs (5.5% [n = 22]). We identified 4 episodes of bacteremia and 1 pelvic abscess (Supplementary Table 1). Only 2 STIs were identified, and neither involved the neogenitalia.

### Microbiology

Urine cultures were available for 87.1% (61 of 70) of those with a UTI. *Escherichia coli* was the most common pathogen (38.5% [27 of 70]), followed by *Klebsiella pneumoniae* (8.5% [6 of 70]) (Table 2). Among UTIs, 12.8% (9 of 70) were treated empirically and 34.2% (24 of 70) were treated despite negative urine cultures. Among SSIs, 86.3% (19 of 22) were treated empirically without wound cultures. Only 13.7% (3 of 22) had wound cultures collected; polymicrobial skin flora grew in 2, and methicillin-susceptible *Staphylococcus aureus* in 1.

**Table 2.** Microbiology and Treatment

Microbiology and Treatment of Post-GAV Infections	Patients, No. (%)
Microbiology of post-GAV infections	
SSTIs (n = 22)	
Polymicrobial	2 (9)
Methicillin-susceptible *Staphylococcus aureus*	1 (4.5)
No wound cultures collected	19 (86.4)
UTIs (n = 70)	
*Escherichia coli*	26 (37.1)
*Klebsiella pneumoniae*	4 (5.7)
*Klebsiella variicola*	1 (1.4)
*Enterobacter cloacae*	1 (1.4)
*Serratia marcescens*	1 (1.4)
*Pseudomonas aeruginosa*	1 (1.4)
Polymicrobial	
*E coli* and *K pneumoniae*	1 (1.4)
*E cloacae* and *K pneumoniae*	1 (1.4)
Unknown^a^	34 (48.5)
BSIs (n = 4)^a^	
*E coli*	4 (100)
Treatment	
SSTIs (n = 22)^b^	
Amoxicillin–clavulanic acid	2 (9.1)
Cephalexin	7 (31.8)
Cefdinir	1 (4.5)
Clindamycin	1 (4.5)
Ciprofloxacin	1 (4.5)
Linezolid	1 (4.5)
Piperacillin-tazobactam	1 (4.5)
Trimethoprim-sulfamethoxazole	12 (54.5)
Vancomycin	1 (4.5)
UTIs (n = 70)	
Amoxicillin–clavulanic acid	1 (1.4)
Cefdinir	1 (1.4)
Cefpodoxime	1 (1.4)
Ceftriaxone	4 (5.7)
Cephalexin	3 (4.2)
Ciprofloxacin	9 (12.8)
Nitrofurantoin	17 (24.2)
Trimethoprim-sulfamethoxazole	28 (40)
Other	4 (5.7)
Unknown	2 (2.8)
BSIs (n = 4)^b^	
Ciprofloxacin	3 (75)
Metronidazole	1 (25)
Trimethoprim-sulfamethoxazole	1 (25)

Abbreviations: BSIs, bloodstream infections; GAV, gender-affirming vaginoplasty; SSTIs, skin and soft-tissue infections; UTIs, urinary tract infections.

^a^Includes cases that were treated empirically without collecting urine cultures or for which culture results were unavailable.

^b^Patients may have received a combination of antibiotics.

*E coli* was isolated in blood cultures of all 4 bloodstream infections. None of the isolates harbored extended spectrum beta-lactamases or carbapenemases (Supplementary Table 2). Identified STIs included primary syphilis and gonococcal urethritis.

### Treatment

Trimethoprim-sulfamethoxazole was the most commonly used antibiotic for UTIs (40% [28 of 70]), followed by nitrofurantoin (24.2% [17 of 70]) and ciprofloxacin (12.8% [9 of 70]) (Table 2). Trimethoprim-sulfamethoxazole (54.5% [12 of 22]) and cephalexin (31.8% [7 of 22]) were the most common antibiotics used for SSIs. All bacteremia episodes were initially treated with intravenous antibiotics before switching to oral antibiotics. Of all patients with UTIs and SSIs, 8.5% and 18% were associated with an emergency department visit, respectively, and 10% and 36%, respectively, were associated with hospitalization within 7 days of diagnosis.

### HIV and PrEP Uptake

Of 398 patients, 16 were living with HIV and prescribed ART at the time of GAV. In the year before GAV, 11 had an undetectable viral load (<20 copies/mL). Of those without known HIV, 86 patients (22.5%) were screened within a year before GAV and only 23 (6%) were prescribed PrEP. An additional 73 patients (19.1%) were screened within a year following GAV, with no new HIV diagnoses.

## DISCUSSION

UTIs and SSIs are the most common infectious complications of GAV, and most occur within a median time (IQR) of 53 (2–162) days following surgery. The incidence of both is similar to those seen in other urogynecologic surgical procedures [9–11]. A notable proportion required an emergency department visit or hospitalization. We did not identify any factors with a statistically significant association with post-GAV infections in our cohort.

The incidence of post-GAV infectious complications reported in the literature varies widely [6, 12, 13]. The incidence of UTI in our cohort (17.5%) is consistent with what has been reported previously. Massie et al [13] reported a UTI incidence of 7% among 117 patients, while Hoebeke et al [14] reported it an incidence of 32% in 31 patients undergoing penile inversion surgery.

Zhao et al [15] reported that SSIs developed in 20.9% of patients undergoing feminizing surgery. In contrast, SSIs occurred in only 5.5% of our cohort. This difference could be explained by newer operative techniques, including robotic and minimally invasive surgical procedures, that contribute to lower infection risk [16, 17]. Data around neovaginal STIs are limited to case reports [18, 19], and compared with cisgender women, transgender women may be at increased risk of acquiring human papillomavirus, HIV, and gonorrhea [20]. In our study, there were no STIs involving the neovagina. Of note, patients were advised to abstain from vaginal or anal sex for 3 months following surgery.

There is no published evidence available to guide surgical prophylaxis in this population. Prior studies have characterized the neovaginal microbiome, especially under the influence of hormone therapy [21, 22]. Birse et al [21] sampled neovaginal secretions and found that anaerobic bacteria like *Prevotella*, *Peptostreptococcus*, and *Porphyromonas* predominated the microbiome, which is unlike cis vaginas with *Lactobacillus* species predominating but very similar to penile foreskin. In our study, gram-negative infections predominated. Whether adding gram-negative coverage to preoperative prophylaxis is indicated requires further study.

We identified a significant gap in HIV screening and PrEP prescribing. Compared with other populations, transgender women have a higher burden of HIV infection [23]. An estimated prevalence of 42% has been reported in US transgender women, with black people being disproportionately affected [24, 25]. However, rates of HIV testing and PrEP use remain suboptimal in this group [26–29]. Gender-affirming healthcare appears to positively affect viral suppression in people living with HIV, with improved access to care [30]. Transgender surgery programs should implement targeted strategies like routine HIV screening and PrEP and ART referrals to provide comprehensive care to a vulnerable population.

Our study has a few limitations. Retrospective record review was used to identify infections. While we used the Centers for Disease Control and Prevention's definition for SSI, documentation of abnormal findings may be incomplete and can result in misclassification. However, a secondary review by an ID physician was performed to optimize outcome classification. UTIs were treated without a urine culture in 12.8% of cases, limiting microbiologic evaluation, and 34.2% had negative cultures, suggesting that these may not be true infections and were likely overtreated. To avoid overestimating UTI incidence, we excluded asymptomatic bacteriuria and used documented symptoms to guide diagnosis. Patients may have sought care at outside institutions for postoperative complications leading to missed infection diagnoses.

We had a small number of infections, which affected our ability to evaluate factors associated with infection. This should be explored further in studies with larger data sets. Finally, we did not examine whether PrEP prescriptions were filled by the patient. This could lead to an overestimation of PrEP use.

Our study adds to the limited available data regarding infectious complications of GAV. Providers should be aware of the risk of postoperative UTIs and SSIs as the demand for GAS increases. More studies are needed to identify risk factors for infections in patients undergoing GAV. In addition, HIV screening and PrEP in this high-risk population remain an urgent priority. Further research is needed to guide antibiotic therapy and stewardship efforts in this population.

## Supplementary Material

ofae526_Supplementary_Data

## Supplementary Data

Supplementary materials are available at *Open Forum Infectious Diseases* online. Consisting of data provided by the authors to benefit the reader, the posted materials are not copyedited and are the sole responsibility of the authors, so questions or comments should be addressed to the corresponding author.
