# Supplementary figures and images for: Infections Following Gender-Affirming Vaginoplasty: A Single-Center Experience
Source: Open Forum Infect Dis. 2024 Sep 11;11(10):ofae526. doi: 10.1093/ofid/ofae526 (PMC11474599; doi:10.1093/ofid/ofae526)

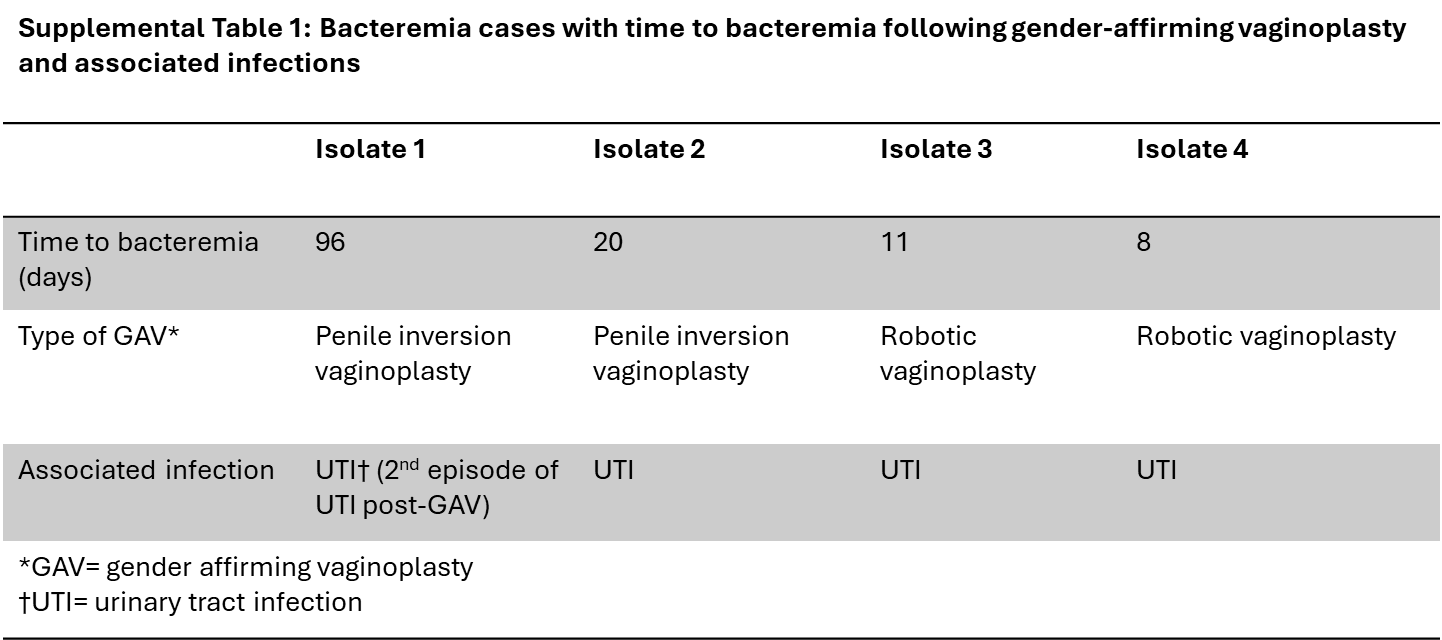


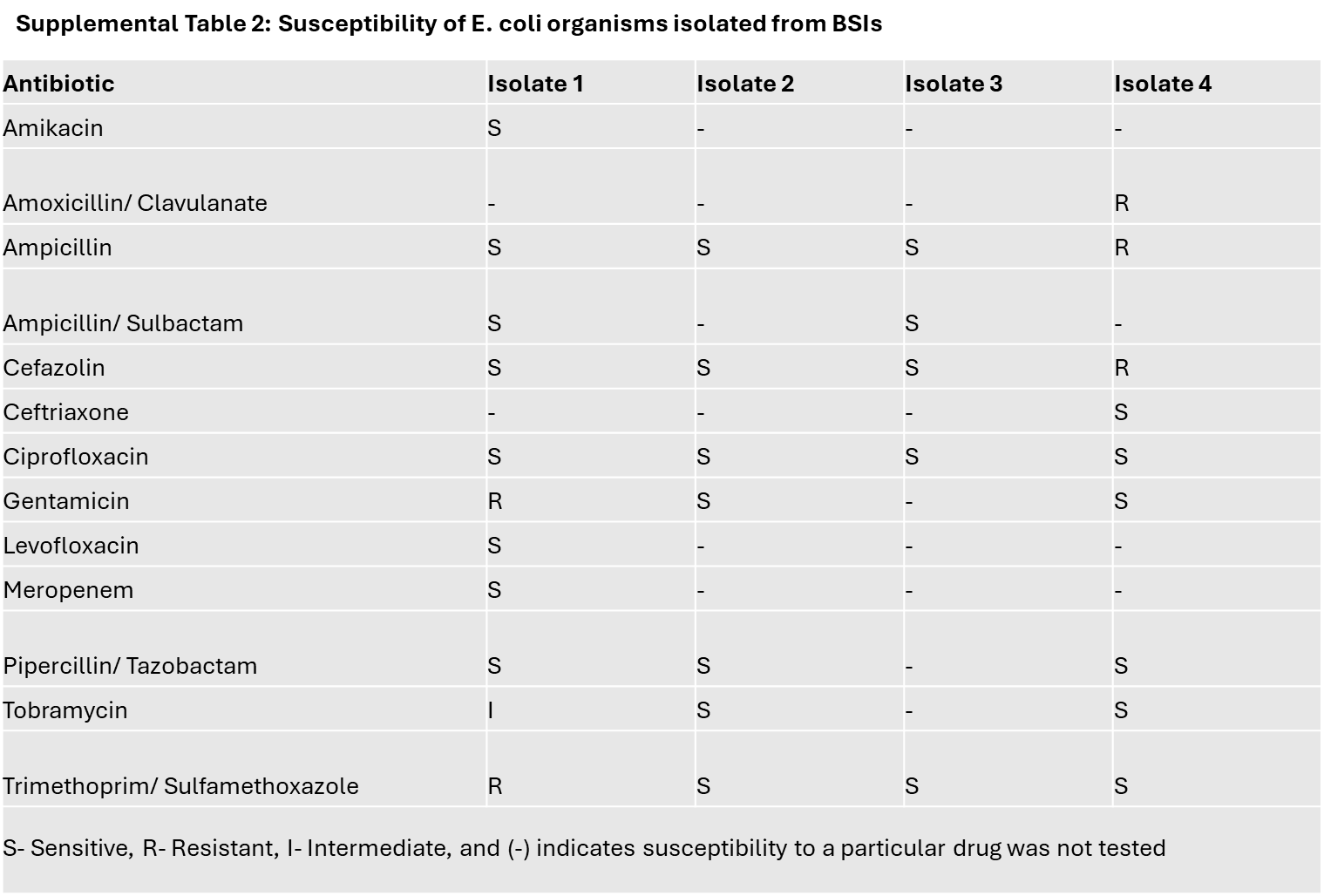

Supplement: ofae526_Supplementary_Data [file ofae526_supplementary_data.docx]
